# Successful Treatment of Recurrent Earlobe Keloid Through Removal of Concealed Underlying Epidermoid Cysts: A Case Report

**DOI:** 10.7759/cureus.44602

**Published:** 2023-09-03

**Authors:** Ju Hyeon Yi, Jung Won Park, Joon-Goon Kim, Byung Ho Oh, Jin Woong Jung

**Affiliations:** 1 Department of Medicine, Yonsei University College of Medicine, Seoul, KOR; 2 Department of Dermatology, Cutaneous Biology Research Institute, Yonsei University College of Medicine, Seoul, KOR

**Keywords:** recurrence, surgery, epidermoid cyst, keloid, ear

## Abstract

In modern clinical practice, earlobe keloids demonstrate a high cure rate through surgical intervention and suitable adjuvant therapies. Furthermore, the concurrence of keloids and epidermoid cysts is uncommon, potentially attributed to the lack of skin appendages within keloid tissue. This case report presents the successful treatment of a recurrent earlobe keloid through the removal of concealed underlying epidermoid cysts. The lesion recurred even after the second excision and proper adjuvant treatments. It was finally stabilized following the removal of epidermoid cysts within the earlobe at the third surgical procedure. These findings emphasize the importance of identifying underlying conditions associated with keloids and addressing inflammation, as these factors significantly influence treatment outcomes and resistance.

## Introduction

In contemporary practice, earlobe keloids exhibit a high cure rate with surgical intervention and suitable adjuvant therapies [1]. Additionally, the coexistence of keloids and epidermoid cysts is rare, possibly due to the absence of skin appendages within keloid tissue [2]. Here, we present a case of recurrent earlobe keloid, which persisted despite multiple surgical interventions and appropriate adjuvant therapies. Notably, successful keloid remission was achieved during the third surgical procedure by identifying and removing multiple minute epidermoid cysts.

## Case presentation

**Figure 1:**
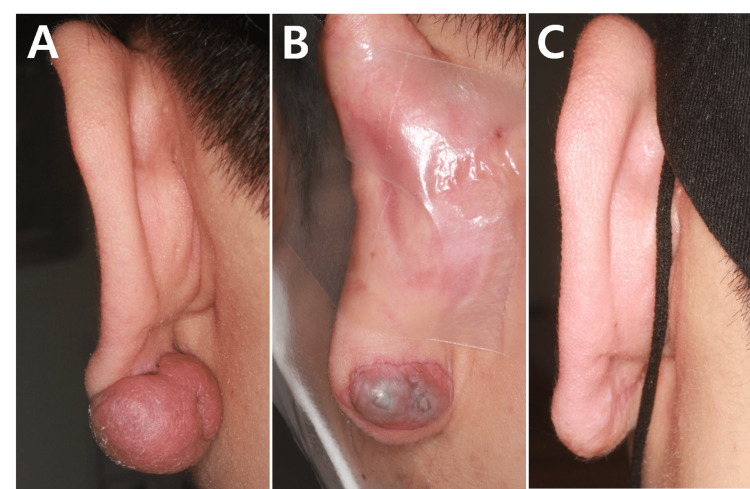
Clinical pictures of the earlobe keloid (A) The initial visit (B) The suspected recurrence of the keloid six months after the second excision (C) 18 months after the last excision

A 26-year-old male presented with a recurrent keloid on the left earlobe. The patient previously underwent surgical excision of the keloid without adjuvant therapy at another hospital’s otorhinolaryngology department one year prior, but the lesion recurred. At the initial visit to our department, the keloid measured 1.2 × 0.8 × 0.6 cm for the anterior portion and 3.2 × 1.8 × 1.5 cm for the posterior portion (Figure 1A). The patient had a history of ear piercing and complained of intermittent pruritus on the lesion. The lesion was surgically removed under local anesthesia, involving excision of the keloid mass and redundant skin tissue. Subsequently, monthly intralesional injections of triamcinolone acetonide (20 mg/mL), pulse-dye laser treatment (Vbeam Perfecta, Candela Laser Corporation, Wayland, MA, USA; spot size: 7 mm, energy fluence: 8-10 J/cm^2^; pulse duration: 3-10 msec, one pass), and compression therapy (with pressure earring) were initiated [3].

Despite adjuvant treatments, the lesion recurred within six months. Subsequent re-excision revealed histopathological evidence of fibrosis accompanied by dilated vasculature. To address the vascular component in the deeper region, an additional intervention was introduced, involving monthly application of a long-pulse 1064 nm Nd:YAG laser (Clarity, Lutronic Corporation, Goyang, South Korea; spot size: 2 mm; energy fluence: 240 J/cm^2^; pulse duration: 30 msec, one pass) in conjunction with the previous adjuvant treatments.

**Figure 2:**
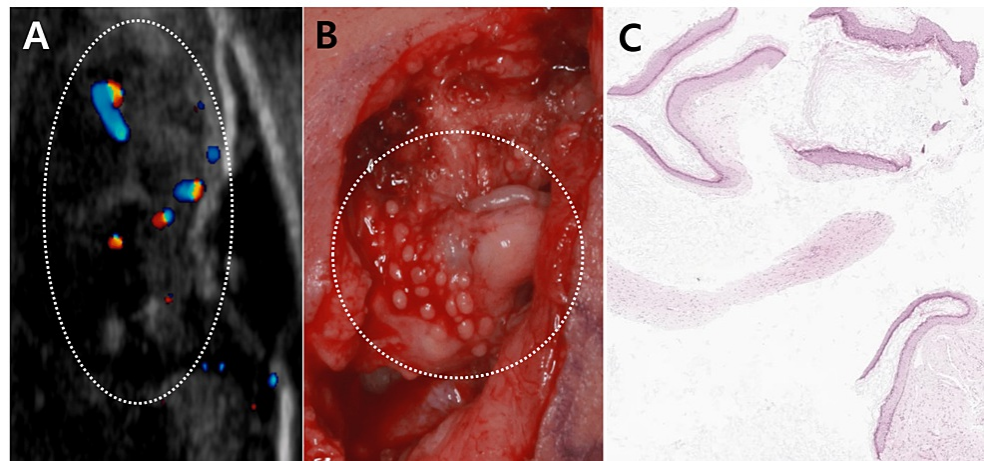
Clinical, radiologic, and histopathologic pictures (A) Ultrasonography reveals multiple hypoechoic and heterogeneous lesions (B) Intraoperative view during the last excision: multiple cyst-like lesions intricately connected with scar tissue are evident (C) Histopathologic features: multiple cysts lined by squamous epithelium and filled with lamellated keratin (H&E, ×40)

During the subsequent six-month follow-up, the patient exhibited a predisposition to recurrence whenever compression therapy was discontinued. This was accompanied by pruritus, erythema, and intermittent swelling, suggestive of underlying inflammation (Figure 1B). On ultrasonography, multiple hypoechoic heterogeneous lesions were identified (Figure 2A). Due to the suspicion of multiple cyst-like formations, surgical removal of the lesion was undertaken.

Throughout the excision procedure, multiple cyst-like lesions intricately connected with scar tissue were discovered and carefully dissected (Figure 2B). Pathologic examination confirmed them as epidermoid cysts (Figure 2C). Post-surgery, the patient received an intralesional triamcinolone injection and laser treatment. In order to reduce inflammation of pilosebaceous follicles, the patient was prescribed a regimen of isotretinoin (30 mg daily) for three months, followed by minocycline (200 mg daily) for six months. No recurrence of epidermoid cysts or keloid overgrowth was observed during the 18-month follow-up period subsequent to the final surgical procedure (Figure 1C).

## Discussion

Epidermoid cysts are benign encapsulated subepidermal nodules filled with keratin, representing the most prevalent form of cutaneous cysts. The cyst’s lining comprises stratified squamous epithelium, leading to the accumulation of keratin layers through desquamation within the cyst [4]. While malignant transformation is rare, approximately 1% of epidermoid cysts have been reported to undergo changes into squamous cell carcinoma or basal cell carcinoma [5]. These cysts usually arise from the blockage of the follicular orifice, originating from the follicular infundibulum [4]. Therefore, the concurrent presence of epidermoid cysts with keloids is uncommon, given that keloids consist of fibrous tissue devoid of skin appendages [2].

Although discovered during the third surgical intervention, the exact onset time of the epidermoid cyst in this case remains uncertain. Since the periauricular area is frequently implicated in cyst formation, it is plausible that the development of cysts could be attributed to infections around pilosebaceous follicles before the formation of the keloid [6]. In that case, the cysts may not have been detected during the previous surgical procedure. Epidermoid cysts may also result from traumatic incidents and subsequent epithelial implantation [7]. The pruritus often induced by keloids may trigger the implantation of the epithelium into the skin, and iatrogenic implantation during surgery could also play a role [4, 8].

A pressure earring comprises two transparent plastic plates connected by plastic screws. Patients are instructed to tighten the screws until initial blanching occurs [3]. Patients self-adjust the earring’s pressure in their daily routine. Consequently, there exists a possibility of excessive pressure, which could potentially precipitate cyst rupture. Ruptured cysts dislocate keratin into the dermis, eliciting inflammatory responses. Chronic inflammation and neovascularization play pivotal roles in the pathogenesis of keloids [9]. Moreover, the mechanical tension resulting from the volume of cysts could potentially contribute to unregulated fibroblast proliferation and collagen accumulation [10].

The lesion exhibited recurrence despite undergoing multiple surgical excisions and receiving adequate adjuvant treatment, including intralesional triamcinolone injection, laser treatment, and compression therapy. However, the lesion was finally stabilized following the removal of epidermoid cysts within the earlobe. Thus, when facing recurrent keloids, especially on the earlobe, it is important to identify underlying conditions that sustain continuous inflammation. Radiological examination, such as ultrasonography, could be valuable for diagnosing underlying cysts [11].

## Conclusions

In conclusion, the identification of concurrent epidermoid cysts during the third surgical intervention proved to be a pivotal factor in achieving successful keloid remission in this case. This underscores the significance of uncovering underlying conditions associated with keloids and addressing inflammation, which can significantly influence treatment outcomes and resistance.
